# 4069 Examining the Effects of A Hybrid Communication Coaching Intervention on Fathers’ Responsive Strategy Use with Children with Autism Spectrum Disorder

**DOI:** 10.1017/cts.2020.127

**Published:** 2020-07-29

**Authors:** Michelle Flippin

**Affiliations:** 1 University of Rhode Island

## Abstract

OBJECTIVES/GOALS: This investigation aimed to mitigate barriers to father involvement in communication intervention for children with ASD and contribute to clinical practice by examining the effects of a hybrid parent coaching intervention for fathers of children with ASD that is tailored to fit both father’s interaction and communication styles as well as individual child characteristics. The Hybrid Father Communication Coaching (HFCC) combined online parent coaching lessons with in-person father-child aquatics sessions in order to increase father’s use of responsive verbal and play strategies. Distal effects on child communication were also investigated. METHODS/STUDY POPULATION: A single subject, multiple baselines across strategies experiment was conducted with one dyad (i.e., father, child with ASD). In the present study, a hybrid father coaching model was used. Parent communication coaching sessions were delivered online, and weekly, father-child aquatics sessions were conducted in person, to provide opportunities for the father to use three targeted responsive strategies (i.e., follow-in comments, follow-in directives, responsive object play) during father-child physical activity. Collateral measures of child communication skills were also investigated. Single subject designs are particularly suitable for autism interventions, as they allow for experimental control with participants who are from heterogeneous populations (McReynolds and Kearn, 1983). The child participant was 5 years, 6 months at the start of intervention and had previously received a community diagnosis of ASD. Throughout the duration of the study, the child attended full-time kindergarten and received in-school speech-language therapy, as a well as 18-20 hours per week of Applied Behavioral Analysis intervention, occupational therapy, physical therapy and speech-language therapy after school. The participating father was a biological parent who resided with the child continuously since birth. The participating father had no other formal parent training in communication intervention before participating. RESULTS/ANTICIPATED RESULTS: The hybrid father communication coaching program (HFCC) yielded positive results for both father and child participant. The father quickly achieved a high level of competency using two of the three, targeted strategies (i.e., follow-in comments, follow-in directives). However, use of a third strategy (i.e., responsive object play) was not maintained above baseline levels. The follow-in comments strategy was used by the participating father more frequently than the follow-in directives strategy. Small increases were documented for child use of spontaneous single words across intervention phases and increased single word use over was maintained eight weeks following intervention. DISCUSSION/SIGNIFICANCE OF IMPACT: The present study provided information regarding the efficacy of a clinically relevant hybrid parent-coaching program, tailored to both father and child characteristics, to enhance fathers’ use of responsive strategies and increase communication skills for children with ASD.

